# Supplementary material for: Factor XIII Activity Might Already Be Impaired before Veno-Venous ECMO in ARDS Patients: A Prospective, Observational Single-Center Cohort Study
Source: J Clin Med. 2021 Mar 14;10(6):1203. doi: 10.3390/jcm10061203 (PMC7999955; doi:10.3390/jcm10061203)
Supplement: Supplementary file 1 [file jcm-10-01203-s001.pdf]

Supplementary Material

Table S1. Standard Laboratory Data during ECMO treatment.

	**T-1 (*n* = 14)**	**T 0 (*n* = 20)**	**T 1 (*n* = 20)**	**T 3 (*n* = 19)**	**T 7 (*n* = 15)**
**Hemoglobin (mg/dl)**	10.1 (8.6/13)	8.85# (8.25/10.1)	9.15 (8.4/10.1)	8.6 (8.3/9.1)	8.7 (8.2/9.7)
**Platelet count (/nl)**	145 (128/210)	126# (94/188) p = 0.0029	126 (79/207)	109 (78/150)	102# (65/135) p = 0.0056
**Leucocyte count (/nl)**	16.7 (8.7/21.3)	12.2# (6.7/16.2)	10.4 (8.2/15.9)	10.5 (8.1/14.7)	13.5 (8.4/16.8)
**Prothrombin Time (%)**	82 (72/88)	74 (63/87)	78 (61/90)	78 (71/95)	77 (64/90)
**aPTT (sec)**	32 (29/35)	32 (29/41)	36 (30/42)	34 (30/40)	37 (36/41)
**Fibrinogen (mg/dl)**	465 (366/584)	433 (307/633)	416 (275/567)	406 (233/602)	329 (223/681)
**AT (%)**	53 (47/58)	43 (37/66)	47 (38/86)	59* (47/84)	85* (60/103)

# indicates significant difference T-1 vs. T 0 , * indicates significant difference compared with T 0.

Table S2. Standard laboratory data after the termination of ECMO treatment.

	**Before Termination of ECMO (*n* = 10)**	**T 1 Post ECMO (*n* = 10)**	**T 3 Post ECMO (*n* = 10)**
**Hemoglobin (mg/dl)**	8.3 (8.2/8.5)	8.0 (7.3/8.6)	8.0 (7.4/8.4)
**Platelet count (/nl)**	95 (44/151)	134 (90/153)	232 (139/331)*# p = 0.0125/0.0166
**Leucocyte count (/nl)**	9.3 (8.5/11.9)	11.1 (8.7/12.3)	9.5 (8.8/9.8)
**Prothrombin Time (%)**	81 (68/87)	74 (59/87)	81 (73/89)
**aPTT (sec)**	39 (35/40)	33 (29/37)	30 (28/34)* p = 0.0125
**Fibrinogen (mg/dl)**	248 (207/527)	349 (231/485)	491 (285/636)# p = 0.0077
**AT (%)**	79 (60/91)	88 (82/99)	93 (79/125)

*indicates significant difference compared with last value before decannulation; # indicates significant difference compared with T1 Post ECMO; $p < 0.0167$.

Table S3. Changes in rotational thrombelastometry during ECMO treatment.

Parameter		T-1 (*n* = 11)	T 0 (*n* = 20)	T 1 (*n* = 20)	T2 (*n* = 19)	T3 (*n* = 19)	T7 (*n* = 15)
**EXTEM**	CT (sec) (38–79)	78 (76/93)	73 (66/89)	78 (68/91)	79 (69/95)	83 (75/99)	87 (77/103)
	CFT (sec) (34–159)	70 (46/103)	73 (59/98)	83 (61/156)	82 (63/171)*	81 (61/120)	94 (82/161)
	A 10 (mm) (43–65)	59 (53/75)	60 (55/66)	57 (43/66)	58 (42/64)*	56 (50/66)	53 (44/59)*
	MCF (mm) (50–72)	67 (62/80)	68 (61/71)	63 (55/71)	65 (53/70)*	64 (57/72)	61 (56/67)
**INTEM**	CT (sec) (100–240)	223 (195/300)	202 (172/223)	212 (176/267)*	216 (174/257)	205 (170/265)	234 (205/279)*
	CFT (sec) (30–110)	78 (52/106)	73 (59/89)	80 (64/147)*	80 (59/164)	82 (61/143)	92 (81/151)*
	A 10 (mm) (44–66)	58 (49/66)	57 (53/64)	55 (42/61)	54 (42/62)	56 (44/65)	52 (42/57)
	MCF (mm) (50–72)	65 (59/72)	64 (59/69)	62 (53/67)*	62 (52/68)	62 (54/71)	61 (52/65)*
**FIBTEM**	CT (sec)	80 (66/100)	67 (60/78)#	75 (60/82)	71 (61/82)	72 (62/88)	83 (71/96)*
	CFT (sec)	126 (62/1001)	219 (91/449)	164 (82/420)	122 (77/293)	135 (93/268)	165 (114/436)
	A 10 (mm) (7–23)	18 (13/31)	20 (15/25)	20 (11/25)	21 (12/30)	22 (16/29)	21 (18/32)
	MCF (mm) (9–25)	18 (14/36)	23 (17/28)	22 (13/29)	24 (13/35)	26 (18/32)	25 (20/36)
**HEPTEM**	CT (sec) (100–240)	186 (180/233)	173 (161/209)	202 (165/233)	200 (165/233)	208 (181/237)	239 (201/260)
	CFT (sec) (30–110)	78 (53/108)	85 (65/102)	87 (62/199)	86 (60/138)	89 (64/112)	99 (91/112)
	A 10 (mm) (44–66)	57 (49/67)	53 (50/62)	52 (35/61)	51 (43/61)	55 (43/61)	51 (44/58)
	MCF (mm) (50–72)	64 (57/73)	60 (55/67)	59 (48/66)	60 (52/66)	62 (52/67)	59 (53/68)

significant difference T-1 vs. T 0 ($p < 0.0083$), * significant difference compared with T 0 ($p < 0.0083$).
